# Crystal Structure of the Full-Length Feline Immunodeficiency Virus Capsid Protein Shows an N-Terminal β-Hairpin in the Absence of N-Terminal Proline

**DOI:** 10.3390/v9110335

**Published:** 2017-11-09

**Authors:** Christelle Folio, Natalia Sierra, Marie Dujardin, Guzman Alvarez, Christophe Guillon

**Affiliations:** 1 Equipe Rétrovirus et Biochimie Structurale, Université de Lyon, CNRS, MMSB, UMR 5086 CNRS/Université de Lyon, IBCP, Lyon 69367 CEDEX 07, France; folio.christelle@gmail.com (C.F.); marie.dujardin@ibcp.fr (M.D.); 2 Laboratorio de Moléculas Bioactivas, Centro Universitario Regional Litoral Norte, Universidad de la República, Paysandú 60000, Uruguay; nataliasierraben@gmail.com (N.S.); guzmanalvarezlqo@gmail.com (G.A.)

**Keywords:** feline immunodeficiency virus, FIV, capsid protein, crystal structure

## Abstract

Feline immunodeficiency virus (FIV) is a member of the Retroviridae family. It is the causative agent of an acquired immunodeficiency syndrome (AIDS) in cats and wild felines. Its capsid protein (CA) drives the assembly of the viral particle, which is a critical step in the viral replication cycle. Here, the first atomic structure of full-length FIV CA to 1.67 Å resolution is determined. The crystallized protein exhibits an original tetrameric assembly, composed of dimers which are stabilized by an intermolecular disulfide bridge induced by the crystallogenesis conditions. The FIV CA displays a standard α-helical CA topology with two domains, separated by a linker shorter than other retroviral CAs. The β-hairpin motif at its amino terminal end, which interacts with nucleotides in HIV-1, is unusually long in FIV CA. Interestingly, this functional β-motif is formed in this construct in the absence of the conserved N-terminal proline. The FIV CA exhibits a *cis* Arg–Pro bond in the CypA-binding loop, which is absent in known structures of lentiviral CAs. This structure represents the first tri-dimensional structure of a functional, full-length FIV CA.

## 1. Introduction

Retroviruses are a major concern for public health in humans but also in animals. The feline immunodeficiency virus (FIV) is the causative agent of an acquired immunodeficiency syndrome (AIDS) in felines [1] with a prevalence rate of up to 30% of domestic cats in some areas [2,3]. Feline immunodeficiency virus is a member of the genus Lentivirus from the Retroviridae family [4], which also contains human immunodeficiency virus (HIV), equine infectious anemia virus (EIAV), and simian immunodeficiency virus (SIV), among others. Due to their common biological characteristics such as virion morphology, physiology, and pathogenesis, FIV has been described as a useful non-primate model for HIV infection, antiretroviral therapy and vaccine development. Feline immunodeficiency virus could also be used as a simple model for a rational drug design for HIV [5,6,7].

Like all infectious retroviruses, the FIV genome contains the three genes—*Gag*, *Pol*, and *Env*—encoding for the structural proteins, the viral enzymes, and the envelope proteins, respectively [8]. The Gag polyprotein is involved in the architecture of the viral particle [9,10]. As for HIV-1 and SIV (but not EIAV), the FIV Gag protein is myristoylated at its N-terminus [11,12,13,14], which allows its targeting of the plasma membrane at the time of particle assembly [15]. During the maturation step of the virus life cycle, the FIV Gag polyprotein is cleaved by the viral protease into different subunits—the matrix protein (MA), the capsid protein (CA), and the nucleocapsid protein (NC)—with a spacer peptide p1 between the CA and NC domains and a C-terminal peptide p2 [16]. The MA protein forms a layer that underlies the viral envelope of the virion, which contains the CA cone-shaped core and the complex formed by the NC and the RNA viral genome [5].

Retrovirus assembly is a critical step in the viral replication cycle and this process is driven by the CA protein [17,18,19]. Indeed, CA protein forms a protective coat around the viral genome and its assembly into a cone-shaped core is characteristic of a mature lentivirus. Moreover, a virus particle without a properly assembled cone-shaped core appears to be non-infectious [20]. Based on this observation, CA protein is a promising therapeutic target for antiretroviral therapy against HIV-1 [21].

Previous crystallographic studies have determined the structure of full-length retroviral CA units as a dimer for EIAV [22] and dimer, pentamer, or hexamer for HIV-1 [23,24,25,26]. Such high-order oligomers are necessary for the formation of a mature capsid core in the viral particle [27,28]. Cross-linking agents and antibody fragments have often been used to stabilize CA for crystallographic studies [29] and CryoEM studies have deciphered supramolecular assemblies of tubular and cone-shaped CAs [30]. Despite low sequence homologies, retroviral CA proteins harbor a rather similar α-helical topology, with two domains—the amino-terminal domain (NTD) and the carboxy-terminal domain (CTD)—connected by a flexible linker [22,23]. This linker plays an important role in the relative flexibility of the CA_NTD_ and CA_CTD_ during the assembly of CA proteins into pentamers or hexamers [25,26]. A highly-conserved sequence is observed amongst all retroviral CA proteins, called the major homology region (MHR), while the rest of the sequence is less conserved between retroviral species. This MHR is required for the correct folding and stability of the CA_CTD_ domain and, thus, is essential for viral replication [31,32].

Thus far, only the structure of the CTD domain of FIV CA has been described [33]. To better understand the molecular and structural specificities of this protein, its crystal structure was determined at 1.67 Å resolution and original features when compared with other lentiviral CAs such as HIV-1 and EIAV were observed. Functional consequences will be discussed.

## 2. Materials and Methods

### 2.1. Construction of Recombinant Plasmid Encoding the FIV Capsid Protein

The full-length native FIV capsid protein was amplified by a polymerase chain reaction on the plasmid p34TF10 (Petaluma strain) as described [34]. A truncated form of the CA protein in its C-terminal end of 9 amino acid residues with a mutation of Pro1 in Thr1 (p24EΔCP-T) was then constructed by PCR using the same protocol and a pair of primers, 5′-AGGATCCAATAGAAGGACGA**ACT**ATTCAAACAGT-3′ and 5′-TGAATTCTCATATTTCTTGACAAGCCCTCAAC-3′, where the Pro1Thr mutation and the introduced stop codon are shown in bold and underlined, respectively. The Pro1Thr mutation was introduced to allow the removal of the 6 × His tag by the Factor Xa protease, which removes all the amino acid of the cleavage site, allowing an intact N-terminus of the protein of interest. However, Factor Xa was not active when the first amino acid after the cleavage site was a proline residue. The product was digested with BamHI and EcoRI and then ligated into the BamHI/EcoRI sites of the vector pRSET-B (Invitrogen, Thermo Fisher Scientific, Villebon-sur-Yvette, France) to form the recombinant plasmid pRSET-p24EΔCP-T encoding the FIV CA protein with a 6 × His tag at its N-terminal end.

### 2.2. Expression and Purification of FIV CA Protein

*Escherichia coli* cells (BL2I (DE3) pLysS, Lucigen, Middleton, WI, USA) transformed with pRSET-p24EΔCP-T were grown in Lysogenic broth medium (Sigma-Aldrich, Saint-Quentin-Fallavier, France) supplemented with 50 mg/mL of ampicillin, at 37 °C. Cell density was monitored by measuring the optical density at 600 nm (OD_600_). When cells reached an OD_600_ value between 0.3 and 0.4, the expression of CA protein was induced by the addition of IPTG (Isopropyl-β-d-1-thiogalactopyranoside, Euromedex, Souffelweyersheim, France) to a final concentration of 1 mM. Expression was carried on for an additional 20 h at 25 °C, then cells were harvested by centrifugation and the pellets were stored overnight at −20 °C.

Purification of CA protein was performed by nickel affinity chromatography, as described for the native CA protein [34]. Briefly, the lysate was clarified by centrifugation at 10,000× *g* for 45 min, and the supernatant was filtered through a 0.45 μm membrane. Purification of the protein from the supernatant was done by batch incubation Ni^2+^-TED resin (Macherey-Nagel, Hoerdt, France) followed by loading onto a gravity column. The column was washed three times with LEW buffer (50 mM NaH_2_PO_4_, 300 mM NaCl, pH 8.5), and the elution was then performed with LEW buffer containing 50 mM of imidazole.

The concentration of CA protein was quantified by spectrophotometry at 280 nm, using a Nanodrop (Thermo Fisher). The purity of the protein was evaluated by SDS-PAGE analysis. Buffer exchange, using Vivaspin ultrafiltration devices (10 kD MWCO, Sartorius, Aubagne, France), was performed against HEPES/NaCl Buffer (50 mM HEPES pH 6.5, 100 mM NaCl).

### 2.3. Removal of the 6 × His Tag

To remove the 6 × His tag, purified CA protein in HEPES/NaCl buffer was digested overnight with 16U of Factor Xa (Qiagen, Courtaboeuf, France) per mg of CA protein, at 19 °C. After proteolysis, the tag-free protein was obtained by loading the sample on a Ni-Nitrilotriacetic acid (NTA) centrifugation column (Proteus, Cliniscience, Nanterre, France) according to the manufacturer’s protocol, and collecting the flowthrough. Purified CA proteins were then concentrated to 7 mg/mL using a Vivaspin centrifugal concentrator (10 kD MWCO, Sartorius).

### 2.4. Crystallization of the FIV CA Protein

Screening of crystallization conditions was performed in 96-well plates using a mosquito nanopipette and commercial crystallization screening kits (Hampton Research, Aliso Viejo, CA, USA and Qiagen) with the sitting drop procedure. The FIV CA protein at 7 mg/mL in HEPES/NaCl buffer crystallized in the presence of an equal volume of 0.2 M magnesium sulfate, 20% PEG 4000, 10% glycerol (condition E11 of the Qiagen PEGs II Suite) supplemented with 10% DMSO final. Using these conditions with the hanging drop technique and drops of 1 μL of protein with 1 μL of crystallization conditions, plate-shaped crystals grew within 15 days. Due to the presence of 10% glycerol in the crystallization solution, the cryoprotection step was dispensable and crystals were directly flash frozen in liquid nitrogen prior to data collection.

### 2.5. X-ray Data Collection and Structure Determination

X-ray data were collected at best to 1.67 Å resolution at the European Synchrotron Research Facility (ESRF) beamline ID30-B (Grenoble, France) at 100 K with a wavelength of 0.99187 Å and a PILATUS 6M-F detector. Crystals belonged to a monoclinic space group C2 with cell dimensions a = 122.2 Å, b = 74.6 Å, c = 77.0 Å, α = γ = 90.0°, β = 128.7°. Indexation and scaling were performed using XDS and XSCALE programs [35]. The structure of FIV CA protein was determined by molecular replacement using the program MrBUMP [36] of the CCP4 program suite [37] and the structures of RELIK (Rabbit Endogenous Lentivirus) CA_NTD_ fragment (PDB ID: 2XGU) [38] and FIV CA_CTD_ fragment (PDB ID: 5DCK) [33] as search models. One solution was found with two monomers in the asymmetric unit and an R-factor of 48%. The crystallographic refinement was performed with PHENIX (version 1.12-2829) [39]. A few residues in the β-hairpin, the cyclophilin binding loop and the C-terminal end were built manually using WinCOOT [40] and six molecules of glycerol were positioned in the electron density maps. The structure was refined to a final R_work_ of 19.7% and R_free_ of 24.1%, respectively, and statistics of the X-ray data are showed in Table 1. It showed a good geometry with 98.6% in preferred regions, 1.4% in allowed regions, and no Ramachandran outliers. The omit map around the *cis*-peptide of the CypBL loop was generated using the PHENIX software with the annealing method on residues 88–92 from chain B. Figures were generated using PyMol (Schrödinger, New York, NY, USA) [41].

## 3. Results

### 3.1. Asymmetric Unit and Crystal Packing

The 1.67 Å crystal structure of FIV CA was determined in the monoclinic space group C2 with two monomers in the asymmetric unit. The two chains (chains A and B) are quasi equivalent in structure and can be superposed with a root-mean-square deviation (RMSD) of 0.63 Å on all Cα pairs.

**Figure 1:**
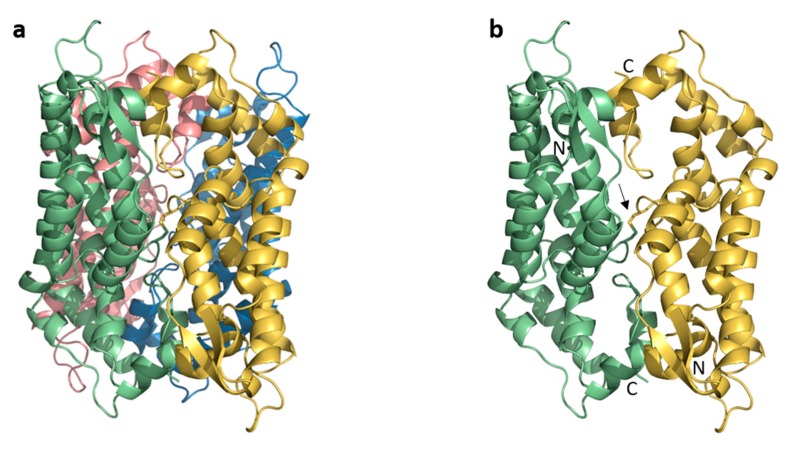
Crystal-packing interfaces and assembly of FIV CA. (**a**) Tetrameric packing of FIV CA protein (chain A: yellow, chain B: green, chain A′: blue and chain B′: pink); (**b**) A-B dimer in the asymmetric unit. The disulfide bridge between chains A and B is labeled with an arrow.

The FIV CA appears to be organized as a dimer of dimers in the crystal (Figure 1a), which assembly is predicted to be stable in solution according to the PISA server for detection of biological oligomers [42]. The A-B dimer (Figure 1b) of the asymmetric unit is related with its symmetrical counterpart A′-B′ via a 2-fold crystallographic axis to form a tetramer A-B-A′-B′ (Figure 1a).

Dimeric CA interfaces have been described for other lentiviruses: for EIAV (PDB ID: 2EIA) [22], the CA_NTD_ from one monomer is interacting with the CA_NTD_ from the second monomer in a head-to-tail orientation, resulting in free CA_CTDs_ in opposite directions in the dimer. Regarding HIV-1 (PDB ID: 3NTE) [43], the CA_NTD_ from one monomer (helices α1 and α2) is interacting with the CA_CTD_ from the other monomer (helices 3_10_ and α9), also resulting in a head-to-tail dimer.

**Figure 2:**
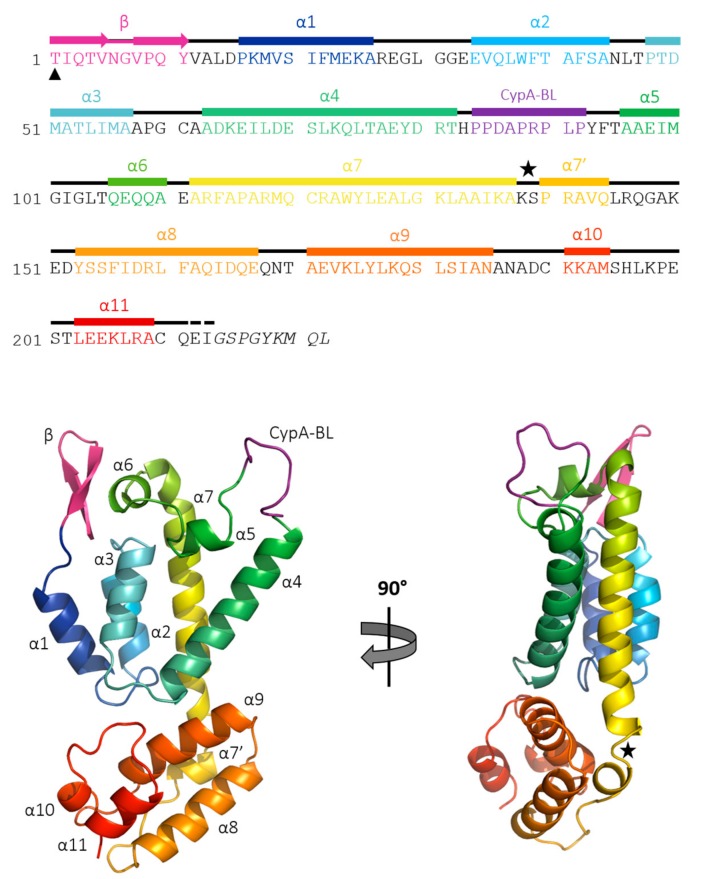
Secondary structures and ribbon diagrams of full-length FIV CA monomer. Helices are colored blue-to-red from N- to C-terminus with helices numbered sequentially from α1 to α11. The β-hairpin is colored in pink and the cyclophilin A-binding loop in purple. The L8 loop, corresponding to the linker between CA_NTD_ and CA_CTD_, is labeled with a black star. The non-native Thr1 is labeled with a black triangle and the truncated residues are written in italic. The two residues not observed in the electron density are labeled with a dashed line.

The A-B dimer of the asymmetric unit contains the two CA monomers in a head-to-tail orientation (Figure 1b) in this FIV CA structure. The FIV CA_NTD_ of one monomer interacts through loop L4 (between α3 and α4, Figure 2) with the loop L12 (between α10 and α11) of the CA_CTD_ of the other monomer. The average buried surface area in this dimer is ~1100 Å^2^ per monomer, with a calculated ΔG of −21.1 kcal/mol. This interface is further stabilized through interactions between the tips of α1 of each FIV CA_NTD_ (Figure 1b). 

Intriguingly, the two monomers in the asymmetric unit are covalently linked by a disulfide bridge between the Cys61 of each CA monomer (Figure 1b). However, SDS-PAGE in non-reducing conditions (Figure S1) and mass spectrometry experiments (data not shown) demonstrate the absence of covalently linked monomers in the protein solution used for crystallogenesis experiments. This suggests that this disulfide bridge is a crystallization artefact, which likely stabilized the dimer. 

### 3.2. The Crystal Structure of Full Length FIV CA

Despite having a low sequence similarity with HIV-1 and EIAV (29% and 39%, respectively), the general fold of FIV CA is similar to that of these lentiviruses with an α-helical, two-domain structure (Figure 2). The FIV CA_NTD_ consists of a 12-residue long β-hairpin followed by 7 α-helices (numbered α1–α7) and contains a cyclophilin-binding loop (CypA-BL in loop L5), which is well defined in the electron density maps. Feline immunodeficiency virus CA_CTD_ follows a short flexible loop (loop L8) linking the two domains, and contains four α-helices (α8–α11), including the highly conserved major homology region (MHR) which is present in all retroviral CA and is essential for viral replication.

### 3.3. Comparisons with Known Structures of Lentiviral CAs

**Figure 3:**
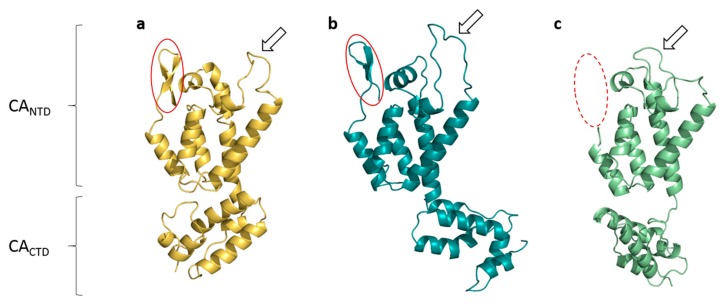
Structural comparison of retroviral capsid protein: (**a**) FIV in yellow; (**b**) HIV-1 in teal (PDB ID: 3NTE [43]) and (**c**) EIAV in green (PDB ID: 2EIA [22]). β-hairpins are circled in solid red when present, in dotted red when not visible in the structures; CypA-BLs are shown with an arrow.

Structural comparisons of FIV CA with full-length HIV-1 and EIAV CAs show that the orientation of the CA_CTD_ with respect to the CA_NTD_ is different in FIV CA (Figure 3). These two domains are close to each other, making the FIV CA structure more compact than what is observed for HIV-1 and EIAV (Figure 3). This feature is due to the small linker between FIV CA_NTD_ and CA_CTD_. Still, CAs_NTD_ and CAs_CTD_ can be superposed separately between FIV, HIV-1, and EIAV with an RMSD between 0.5 and 1 Å on Cα pairs (data not shown), demonstrating the global conservation of the lentiviral CA fold in FIV CA. Similarly, a low RMSD value (0.5 Å) is obtained when superposing our FIV CA_CTD_ with the recently solved FIV CA_CTD_ fragment [33]. 

The first 12 N-terminal residues of FIV CA consist in a β-hairpin motif wherein strands appear longer than that of HIV-1 (5 and 4 amino-acids for FIV CA versus 2 and 3 for HIV-1 CA, Figure 3b). This region is not observed in EIAV CA (Figure 3c). Another difference concerns the CypA-BL of the NTD, which appears to be smaller in this FIV CA structure (Figure 3a, arrow) compared to other retroviral CA proteins such as HIV-1 (Figure 3b). 

**Figure 4:**
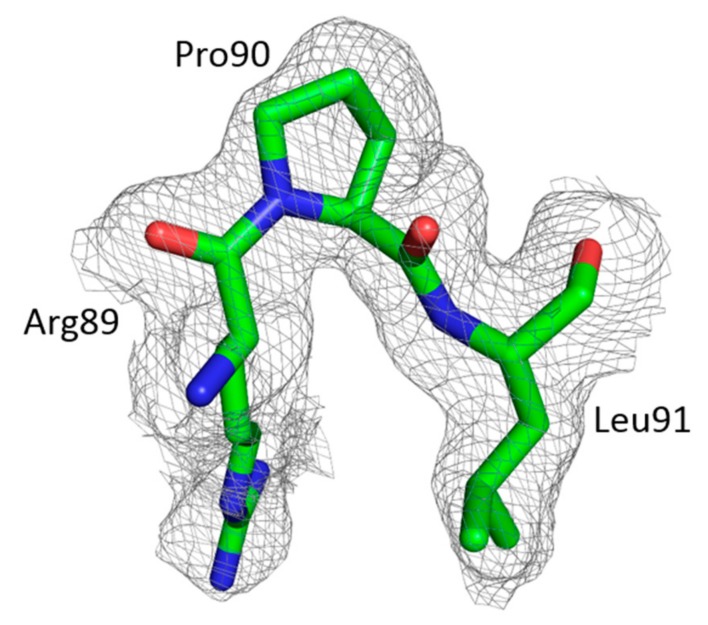
Omit map of FIV CA Arg89-Pro90 *cis*-peptide bond from chain B CypA-BL, contoured at 1σ. The oxygen and nitrogen atoms are shown in red and blue, respectively.

It is worth noting that the CypA-BL in FIV CA contains a *cis* Arg89–Pro90 peptide bond in both chains in the asymmetric unit, which is 100% in *cis*-conformation (Figure 4). Such *cis*-peptides are not observed in the CypA-BL of published HIV-1 or EIAV structures. 

Regarding the CTD, Cys190 and Cys210 show an alternate conformation resulting in the formation of an intramolecular disulfide bridge with an occupancy of about 75%, which is absent in the published structure of FIV CA_CTD_ [33]. The 3_10_-helix which is observed in the HIV-1 CA_CTD_ between α7 and α8 [33] appears to be replaced by an α-helix (numbered α7′) in this FIV CA full-length structure (Figure 2). 

## 4. Discussion

To define the specificity of the molecular mechanisms underneath FIV assembly, investigation of the structure of the FIV full-length CA protein was performed and compared with structures of other retrovirus CA proteins. Feline immunodeficiency virus CA protein is mostly composed of α-helices, like the CA protein of other retroviruses, confirming that the overall α-helical fold of CA protein is highly conserved among retroviruses. 

During the assembly, retroviral CA proteins assemble into pentamers and hexamers [44,45] to form a cone-shaped core, but no pentameric or hexameric assemblies were observed for this FIV CA structure. Nevertheless, six monomeric FIV CAs could be superimposed on a HIV-1 native hexamer (Figure S3), without requiring strong conformational changes. This superposition does not generate any steric clash between FIV monomers. In this superposition, the C-terminal domain of FIV CTD are not completely superimposed to that of HIV-1 in hexamers. This could come from the fact that the dimeric interface of our structure has set the flexible linker between NTD and CTD of FIV in a position which results in a different orientation of the CTD than the one observed in HIV-1 hexamers. However, as isolated CTDs of FIV and HIV-1 can be superimposed with a RMSD of less than 1 Å on Cα pairs, this structure of monomeric FIV CA is compatible with the formation of hexamers as functional units for capsid assembly.

The crystal structure of FIV CA contains one dimer of CA protein (chains A and B) in the asymmetric unit. This dimer is probably not functional since the N-terminal ends are oriented in opposite directions while they should be oriented in the same way for proper interaction with the FIV matrix protein (MA). Dimerization of HIV-1 CA_CTD_ has been described as involving a tryptophan residue at position 184 [46]. Notably, no tryptophan is observed in the CTD of FIV CA. Specific identification of dimeric interfaces will be necessary to understand the specific mechanisms of FIV oligomerization [46].

This study’s structure finds that the FIV CA dimer is covalently linked by a disulfide bridge between the Cys61 of each monomer. It showed that this disulfide bridge is a crystallization artifact, as it is absent from the protein solution used for crystallogenesis. This is consistent with the observation that Cys61 is not involved in disulfide bridges in functional FIV CA [19]. This crystallization artifact is likely due to the presence of dimethyl-sulfoxide (DMSO) in the crystallization condition. Indeed, DMSO has been reported to promote oxidation of thiol into disulfide at low pH and room temperature [47]. The presence of this artifactual disulfide bridge might have helped stabilize the CA dimer in the asymmetric unit during the crystallization process, resulting in the formation of FIV CA crystals which were not obtained in the absence of DMSO (data not shown). However, as a drawback, the formation of this bridge might also have stabilized the FIV CA dimers in non-relevant interfaces and/or impaired the formation of high order oligomers (pentamers, hexamers) which are necessary for the formation of the retroviral capsid. Crystallogenesis experiments in the absence of DMSO are therefore currently pursued to unambiguously identify the functional oligomeric interfaces. 

In addition to this interchain artifactual disulfide bond between Cys61 to each monomer, it was observed that Cys190 and Cys210 form an intramolecular disulfide bridge in 75% of the proteins in the crystal for both chains of the asymmetric unit. Although it was absent from the isolated FIV CA_CTD_ structure [33]. This cysteine bond agrees with biochemical studies who reported that cysteines Cys190 and Cys210 are involved in an intramolecular disulfide bond which is necessary for FIV capsid assembly and FIV infectivity [19]. Moreover, this cysteine bond is highly conserved across several retroviral CA proteins, from HIV-1 to EIAV [22,48]. Thus, this structural feature is probably relevant for the biology of FIV CA. 

As expected from biochemical data [19], the last free cysteine of FIV CA—Cys121 (from α7′)—is not be involved in any cysteine bond. Interestingly, the sulfur atom of this cysteine participates as a cluster with sulfur atoms of Met51 (from α4) and Met100 (from α6). These three sulfur atoms show an intriguing feature, as they are aligned and distributed at 4 Å one after the other (data not shown). This distribution is unique to FIV CA as other retroviral do not harbor a cysteine residue homologous to FIV Cys121, but its function (if any) remains to be determined.

**Figure 5:**
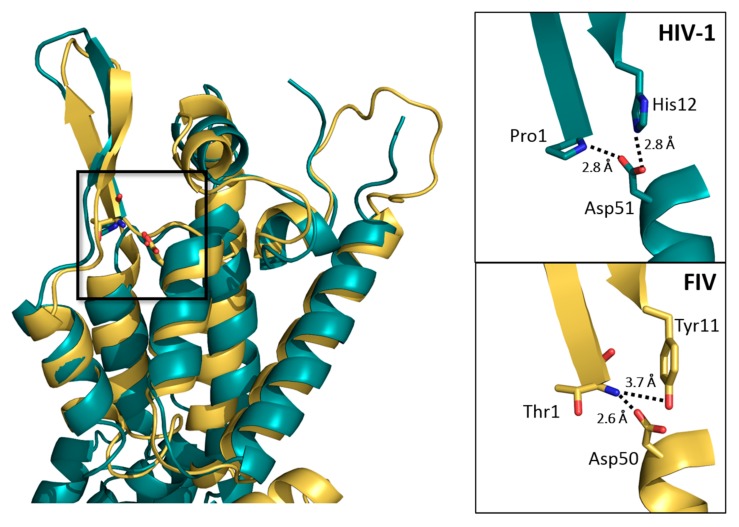
Details of the N-terminal β-hairpin of FIV CA. Superposition of FIV CA_NTD_ (yellow) and HIV-1 CA_NTD_ in its “open” conformation (teal, PDB ID: 5HGL [26]). A detailed view of the boxed region (right) shows the salt bridges of HIV-1 Pro1 and His12 with Asp51 (top) and FIV Thr1 with Asp50 and Tyr11 (bottom).

However, the FIV CA monomeric structure that was obtained harbors important features to understand FIV assembly. An example is that FIV CA contains at its amino-terminal end a β-hairpin motif (Figure 5). This motif could be expected as it has been shown to be required for the formation of the HIV-1 capsid core particle since it participates directly in intermolecular CA–CA interactions [23,49]. This study demonstrates that this β-hairpin seems to adopt a conformation which corresponds to the “open” conformation described for HIV-1 CA protein [26] which might be important for the import of dNTP in the virus core during reverse-transcription. 

The Pro1 of CA has been described as essential for the formation of this N-terminal β-hairpin in HIV-1 CA [50]. Interestingly, this proline is conserved in most lentiviral CA proteins such as HIV-1, SIV, and EIAV, which probably reflects a key function for viral replication. Thus far, the functional role of this proline has been attributed to the formation of the β-hairpin. However, the Pro1 into Thr1 mutation that this study has introduced in FIV CA for practical reasons (described in the Materials and Methods section) did not impair the formation of the β-hairpin nor the assembly of FIV CA in vitro (Figure S2). Moreover, the salt bridge between the terminal NH^2+^ group of Pro1 and the side chain carboxyl group of Asp51, which stabilizes this motif in HIV-1 [50], has an equivalent in this FIV CA structure. Indeed, we could observe a salt bridge between the terminal NH^2+^ group of Thr1 and the side chain carboxyl group of Asp50 (Figure 5), with about the same bond length than that observed for the Pro–Asp salt bridge in HIV-1 CA (2.6 Å versus 2.8 Å, respectively). Studying the functionality of this Pro1Thr CA mutant for FIV or HIV-1 replication is beyond the scope of this article, but could help understanding if the key function of this proline indeed is to induce the formation of the β-hairpin, (which is not suggested by our data) or if its main role is for the Gag precursor to adopt the best conformation at the MA-CA junction for efficient protease cleavage during maturation.

Additionally, amino-acid His12 of HIV-1 CA has been shown to be important for the stabilization of the β-hairpin in an “open” or “closed” position, since it is involved in a salt bridge with Asp51 of helix α3 [26]. The equivalent of His12 in FIV CA structure is a tyrosine (Tyr11), which is not able to form a salt bridge with Asp50 (Figure 5). However, in this study’s structure, a salt bridge exists between the hydroxyl group of this Tyr11 and the terminal NH^2+^ group of Thr1 (3.7 Å, Figure 5). This salt bridge between the extremities of the two strands of the β-hairpin in FIV CA might contribute to enhance its stability.

The CypA-BL present in lentiviral capsid protein is also observed in the FIV CA_NTD_. Interestingly, as for RELIK CA [38] but not HIV-1 CA, the presence of a *cis* Arg89–Pro90 peptide bond (Figure 4) in FIV CA CypA-BL could be detected. Remarkably, among the five prolines present in FIV CypA-BL, only this Pro90 residue is in a *cis*-conformation. The CypA is a *cis*–*trans* peptidylprolyl isomerase with a stronger specificity for natural substrates containing *cis*Pro [51]. This could explain why, among the proline residues of FIV CypA-BL, Pro90 is the critical target for CypA binding to FIV CA [52]. 

The last two residues of the CA_CTD_ domain are not defined in the electron density. This confirms the high flexibility of the C-terminal end of FIV CA protein, which had already been truncated by nine residues to avoid problems with crystallogenesis. This C-terminal end might be flexible to allow the correct conformation of the CA–NC cleavage site of the Gag polyprotein, as was hypothesized for the flexibility of the FIV MA C-terminus in the MA–CA cleavage process [53].

Altogether, these results show that the various domains which have been involved in key functions of retroviral CA, or which have been observed as important for FIV replication, are present in this FIV CA structure, although with their own specificities.

## 5. Conclusions

This study determined the first atomic structure of full-length FIV CA to 1.67 Å resolution. The monomeric FIV CA is a functional capsid protein and displays a standard α-helical CA topology with a short linker between its N- and C-terminal domains. Feline immunodeficiency virus CA also harbors original features like its *cis* Arg89–Pro90 bond, which is visible for the first time in the structure of a retroviral CA. Moreover, despite the absence of the conserved N-terminal proline in this FIV CA construct, the amino-terminal β-hairpin motif is formed in a similar conformation to that of HIV-1 CA, although it is longer in FIV CA. How these features play a role in the differences observed in CA assembly in vitro [30,34] remains to be determined by the elucidation of the structure of high-order oligomers of FIV CA. Altogether, the crystallographic structure of FIV CA represents, in its monomeric form, a functional capsid protein with original features when compared to HIV-1 and EIAV.

## Figures and Tables

**Table 1:** Summary of X-ray data collection and refinement statistics. Values in parentheses are for the highest-resolution shell.

Data Collection	FIV CA
Space group	C121
Unit cell parameters	
a, b, c (Å)	122, 74, 77
α, β, γ (°)	90, 128, 90
Resolution range (Å)	38.5–1.6 (1.75–1.67)
R_sym_ (%)	4.4 (77.6)
I/σI	11.2 (1.0)
Completeness (%)	92.2 (94.1)
Redundancy	2.38
CC (1/2)	99.9 (51.6)
**Refinement**	
Resolution range (Å)	38.5–1.67
Number of unique reflections	60,447
R_work_ (%)	19.7
R_free_ (%)	24.1
Number of proteins atoms	3331
Number of water/glycerol	699/6
Mean B-factor (Å^2^)	28.9
Coordinate deviations	
RMSD bond lengths (Å)	0.007
RMSD angles (°)	0.888
PDB ID	5NA2

## Supplementary Materials

The following are available online at www.mdpi.com/1999-4915/9/11/335/s1.
