# Supplementary material for: Crystal Structure of the Full-Length Feline Immunodeficiency Virus Capsid Protein Shows an N-Terminal β-Hairpin in the Absence of N-Terminal Proline
Source: Viruses. 2017 Nov 9;9(11):335. doi: 10.3390/v9110335 (PMC5707542; doi:10.3390/v9110335)
Supplement: Supplementary file 1 [file viruses-09-00335-s001.pdf]

Article

Crystal Structure of the Full-Length Feline Immunodeficiency Virus Capsid Protein Shows an N-Terminal β-Hairpin in the Absence of N-Terminal Proline

Christelle Folio[1], Natalia Sierra[2], Marie Dujardin[1], Guzman Alvarez[2], and Christophe Guillon[1],*

1 Equipe Rétrovirus et Biochimie Structurale, Université de Lyon, CNRS, MMSB, UMR 5086 CNRS/Université de Lyon, IBCP, Lyon, France

2 Laboratorio de Moléculas Bioactivas, Centro Universitario Regional Litoral Norte, Universidad de la República, Paysandú, Uruguay

* Correspondence: christophe.guillon@ibcp.fr; Tel. : +33(0)4-37-65-29-04

Supplementary Materials

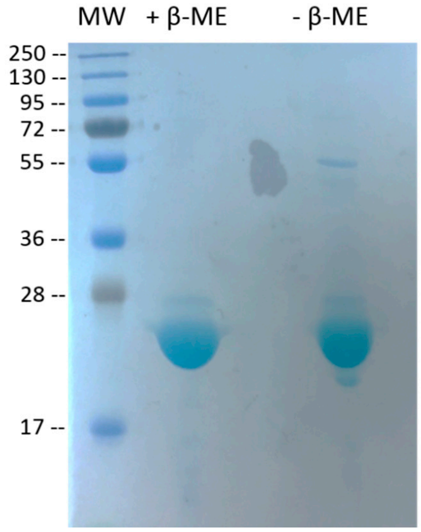

Figure S1: Absence of a disulfide bridge between CA monomers in solution. One microliter of FIV CA protein was loaded in presence (+ βME) or absence (- βME) of reducing agent β-mercaptoethanol on a SDS-PAGE

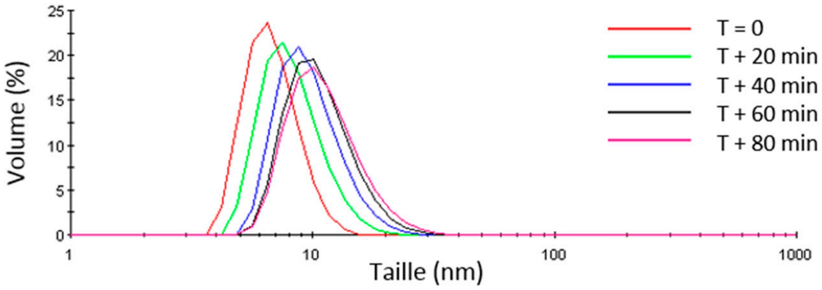

Figure S2: Analysis of FIV CA protein by dynamic light scattering (DLS). Assembly kinetics of FIV p24EΔCP-T protein at 7 mg/mL in presence of NaCl 1M [1].

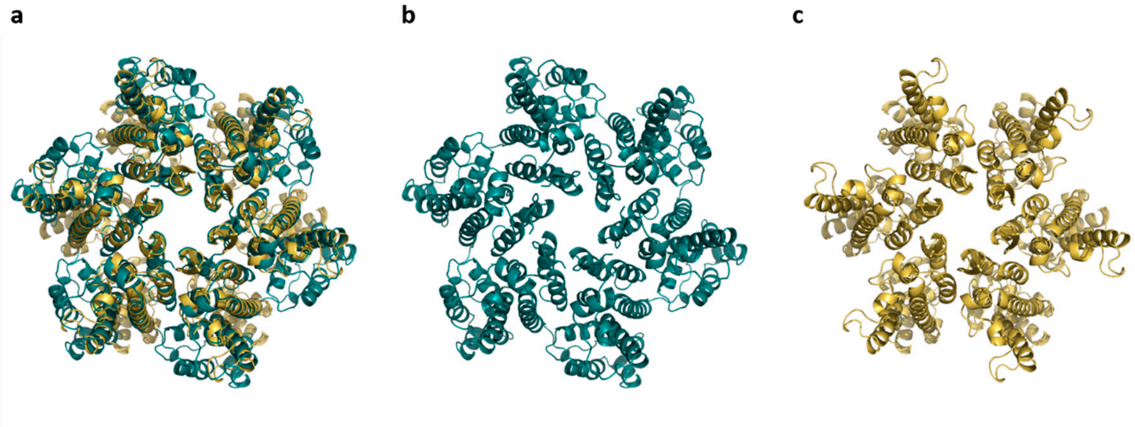

Figure S3: HIV-1 and FIV CAs hexamers. (**a**) Superimposition of 6 FIV CA monomers (yellow) on HIV-1 CA native hexamer (teal, PDB ID: 4XFX [46]) with respect to the NTD. (**b**) HIV-1 CA native hexamer. (**c**) FIV CA reconstructed hexamer.

References :

1. Serriere, J., D. Fenel, G. Schoehn, P. Gouet, and C. Guillon, *Biophysical characterization of the feline immunodeficiency virus p24 capsid protein conformation and in vitro capsid assembly*, PLoS One, 2013. **8**(2): p. e56424.
